# Supplementary material for: Perfusable Tissue Bioprinted into a 3D-Printed Tailored Bioreactor System
Source: Bioengineering (Basel). 2024 Jan 9;11(1):68. doi: 10.3390/bioengineering11010068 (PMC10813239; doi:10.3390/bioengineering11010068)
Supplement: Supplementary file 1 [file bioengineering-11-00068-s001.zip › Supplementary Data.pdf]

Supplementary Data

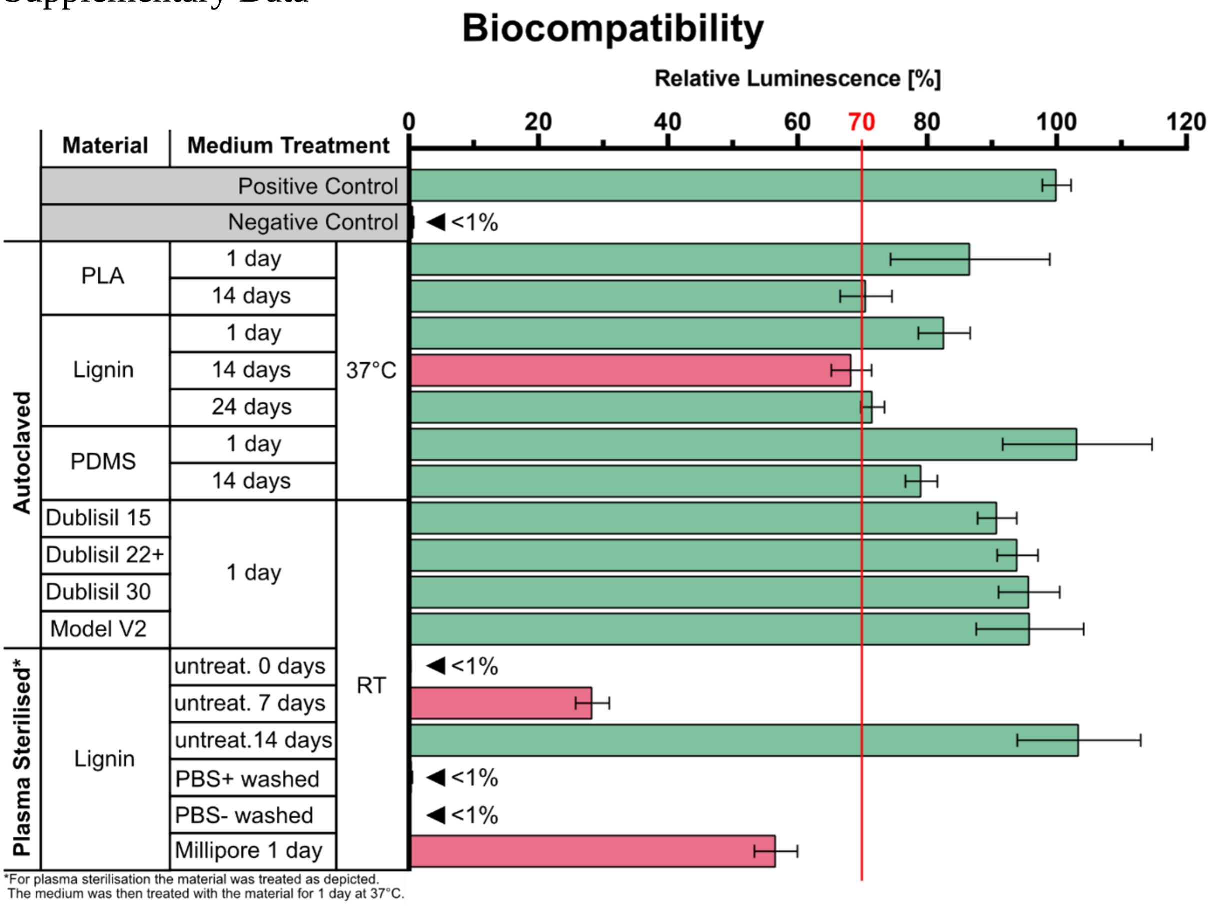

Figure S1: Biocompatibility of several materials used in the bioreactor system using CellTiterGlo assay. A viability of 70% of the positive control is considered to be the threshold to show biocompatibility.

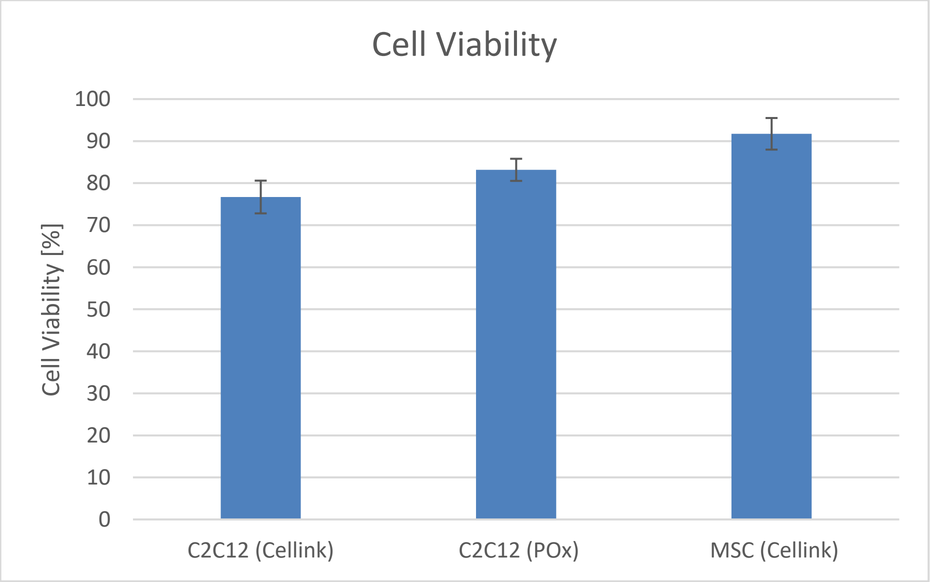

Figure S2: Cell viability of C2C12 cells and MSC after cultivation under dynamic culture conditions for 14 days (C2C12) and 21 days (MSC), respectively. Four representative areas from the Live-Dead Staining were manually counted for each approach.

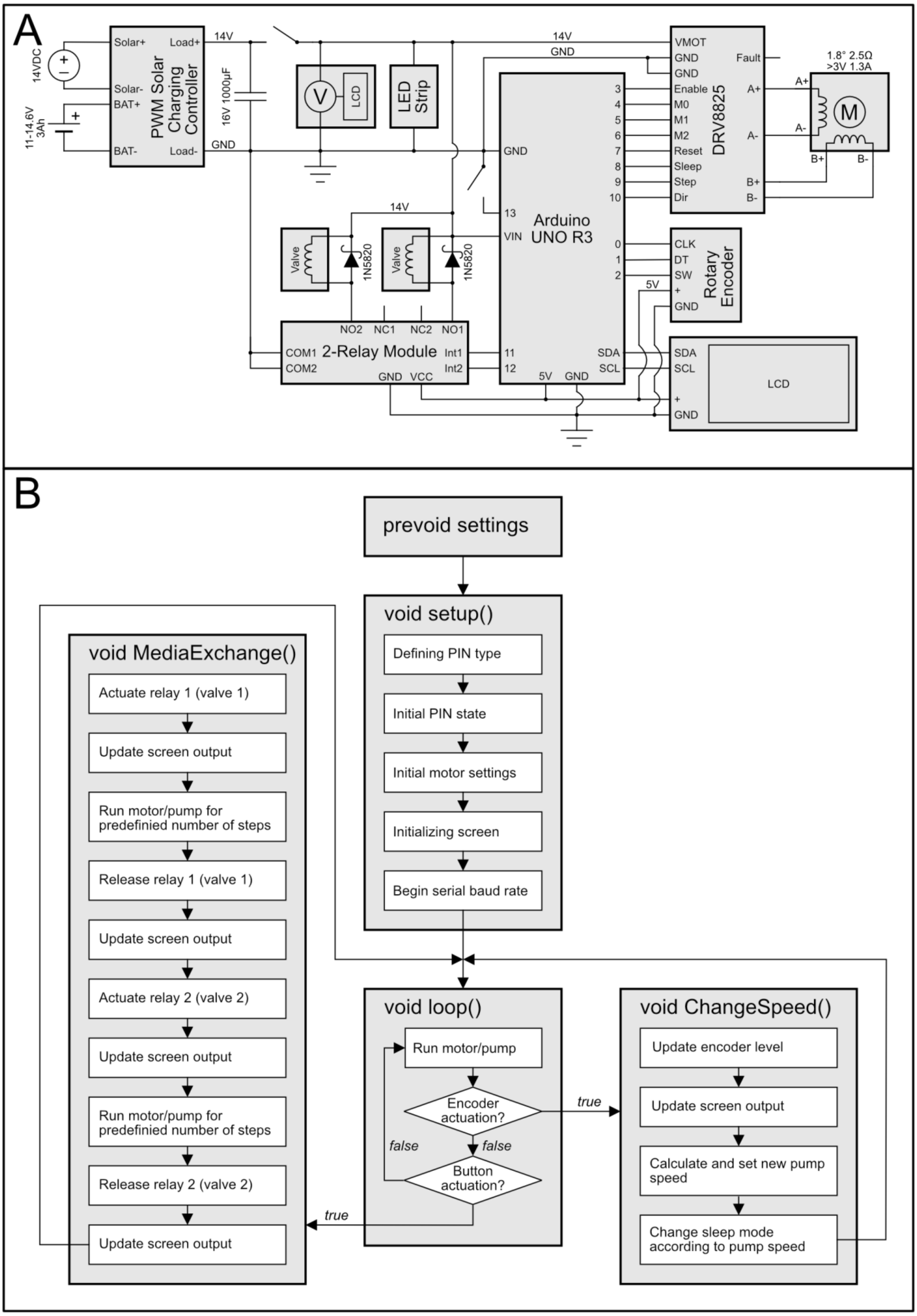

Figure S3: Circuit diagram (A) and flow diagram (B) of the automated bioreactor docking station.
